# Effect of Internal Curing on Early Shrinkage and Crack Resistance of UHPC by SAP and Ceramsite

**DOI:** 10.3390/ma19040806

**Published:** 2026-02-20

**Authors:** Xianqiang Wang, Jinxu Wang, Xiaonan Feng, Zaixin Yang, Jiancheng Gu, Wenqin Deng

**Affiliations:** 1 School of Civil Engineering, Nanjing Tech University, Nanjing 211816, China; 2 State Key Laboratory of Safety, Durability and Healthy Operation of Long Span Bridges, JSTI Group, Nanjing 210019, China; 3 Jiangxi Provincial Transportation Design and Research Institute Co., Ltd., Nanchang 330200, China

**Keywords:** ultra-high performance concrete (UHPC), internal curing, SAP, ceramsite, shrinkage, crack resistance

## Abstract

This study investigated the effects of varying water–binder (w/b) ratios and internal curing materials—superabsorbent polymer (SAP) and ceramsite—on the shrinkage behavior and crack resistance of ultra-high-performance concrete (UHPC). Although internal curing has been extensively studied, the comparative effectiveness of different internal curing materials on early-age shrinkage and restrained cracking behavior of UHPC under consistent mixture proportions remains unclear. To address this gap, a systematic experimental comparison of SAP and ceramsite was conducted. The influences of w/b ratio and different amounts and addition methods (dry and pre-absorbed addition) of SAP and ceramsite on the flowability, mechanical properties, early autogenous shrinkage, drying shrinkage, and early crack resistance of UHPC were discussed. Findings indicate that increasing the w/b ratio reduces autogenous shrinkage but compromises mechanical properties, altering the cracking mode from primary microcracks to a few wider cracks. Pre-saturated ceramsite (less than 10% volume) and SAP effectively mitigate autogenous and drying shrinkage, enhancing crack resistance without significantly reducing mechanical properties. However, exceeding a ceramsite volume dosage of 10% or using the dry addition method increased the flowability of UHPC, while decreasing crack resistance. Microstructural analysis reveals that internal curing materials facilitate hydration and enhance structural density through the formation of ettringite and calcium silicate hydrate. To optimize shrinkage reduction while maintaining mechanical properties, SAP should be incorporated in a dry state, with a dosage limited to 0.4% of the mass of the cementitious material; ceramsite needs to be pre-saturated and limited to 5% of the total volume.

## 1. Introduction

UHPC has garnered significant attention owing to its outstanding mechanical properties and durability [1,2,3]. Widely utilized across various engineering domains, UHPC boasts remarkable characteristics such as extremely low porosity, high strength, and density [4,5]. However, while ensuring high strength, its low w/b ratio and high cementitious material dosage characteristics also lead to low hydration and high shrinkage, rendering it susceptible to cracking [6,7]. Consequently, this diminishes its bearing capacity and durability. Effectively controlling the early shrinkage of UHPC and enhancing its crack resistance has thus emerged as a pivotal focus and challenge in current research.

Currently, promoting the hydration of cementitious materials through high-temperature steam curing is a common method to reduce the early autogenous shrinkage of UHPC [8]. However, this method is highly energy-intensive and impractical for UHPC curing in cast-in-place or complex working conditions. Internal curing is an effective method for concrete curing [9,10]. By introducing water-absorbing materials to form a small water reservoir inside the concrete, as the cement hydrates, a humidity gradient and capillary negative pressure appear inside the concrete, and the internal curing materials release water to inhibit the development of concrete shrinkage [11,12,13]. Common internal curing materials include lightweight aggregates (LWA) [14,15] and SAP [16,17]. The water absorption rate of LWA is 10%~30%, mainly through capillary forces, such as ceramsite [18], zeolite [15], and perlite [19], which are usually saturated before being added to concrete [20]. SAP is a high polymer compound commonly referred to as a hydrogel, showcasing remarkable water absorption capabilities [21,22], capable of absorbing water tens to thousands of times its weight and retaining it within its structure without release [23,24,25].

In order to optimize the internal curing effect of internal curing materials on concrete, some scholars have studied the effects of different internal curing materials on concrete performance. Ghourchian et al. [15] evaluated the internal curing efficiency of natural zeolite and lightweight expanded clay aggregates (LECA) in cement mortars, finding that despite a high water absorption of 15.6%, zeolite aggregates failed to mitigate early-age shrinkage because they retained the majority of absorbed water in nm-sized pores at relative humidity (RH) levels as low as 80%, whereas LECA effectively released water at high RH (>97%) and significantly reduced total shrinkage. Browning et al. [26] investigated the efficacy of prewetted vacuum-saturated (PVS) lightweight aggregate (LWA) as an internal curing agent in concrete, demonstrating that replacing 8.9–13.8% of normalweight aggregate with LWA could reduce drying shrinkage by up to 39% within 30 days of drying, and further finding that combining LWA with a 60% slag cement replacement achieved a peak shrinkage reduction of 91% compared to granite control mixtures. Geiker et al. [24] found that the addition of SAP can significantly improve the shrinkage cracking of concrete. Compared with LWA, SAP has a higher efficiency in inhibiting autogenous shrinkage and the improvement effect of pre-absorbed SAP is more obvious. Lura et al. [27] pointed out that the impact of SAP on the shrinkage properties of cement-based materials is significantly affected by its particle size and dosage; when the SAP dosage is 0.3%, larger SAP particles can lead to more obvious early flowability and help inhibit autogenous shrinkage for a longer period of time. However, when the SAP dosage increases to 0.6%, the effect of SAP particle size on the autogenous shrinkage of cement-based materials is no longer so significant. De Meyst et al. [25] employed corrugated tube and restrained ring tests to evaluate the internal curing efficiency of different SAPs, finding that the addition of 0.2–0.3% poly-acrylate-based SAPs could mitigate autogenous shrinkage by up to 97% in high-performance mortar, although the mitigation efficiency varied significantly depending on the polymer chemistry and dosage (19–89% reduction for sulfonate-based SAPs). Liu et al. [28] utilized IRH sensors and shrinkage measurements to investigate the mitigation effect of 0.6% superabsorbent polymers (SAPs) on ultra-high strength concrete (UHSC), finding that SAPs could reduce drying shrinkage by up to 60.5% in large specimens and maintain the IRH above 90% in inner layers (3–10 cm from the drying surface) within 28 days.

Most existing studies focus on shrinkage strain measurement in a free state, but in actual engineering, UHPC is mostly under constraint conditions, and the risk of cracking under constraint stress and the inhibitory effect of internal curing materials are insufficiently evaluated; at the same time, internal curing materials generally sacrifice mechanical properties in exchange for shrinkage inhibition, and how to achieve strength-shrinkage synergistic optimization through mix design still needs further exploration. Therefore, it is imperative to explore the impact of internal curing methods on the mechanical properties and early shrinkage crack resistance of UHPC to improve structural durability and expand the use scenarios of UHPC projects.

This study primarily used the w/b ratio, the dosage, and the addition method of SAP and ceramsite as key design variables. The water absorption performance of SAP and ceramsite in the filtrate of different cementitious materials was analyzed. It discussed the flowability, mechanical properties, early autogenous shrinkage, internal relative humidity (IRH), and drying shrinkage of UHPC under different mix ratios, as well as the crack resistance. At the same time, SEM was employed to analyze the internal curing mechanism. This study not only quantitatively analyzed characteristics such as early autogenous shrinkage and drying shrinkage, but also combined the flat plate constraint to simulate the shrinkage and cracking behavior of UHPC when constrained in actual engineering, and obtained the optimal dosage of internal curing materials and material processing methods, which has certain practical engineering significance.

## 2. Materials and Methods

### 2.1. Materials

**Figure 1:**
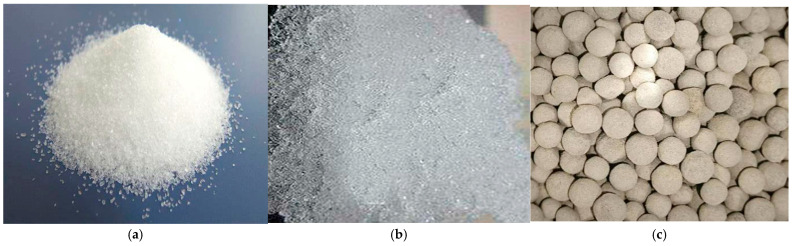
Macroscopic morphology of internal curing materials used in this study: (**a**) superabsorbent polymer (SAP) in dry state; (**b**) SAP after absorbing water; (**c**) ceramsite.

P.O. 42.5 Portland cement was supplied by Anhui Conch Cement Co., Ltd. (Wuhu, China). Class I silica fume and fly ash were provided by Henan Yixiang New Materials Co., Ltd. (Zhengzhou, China). The corresponding chemical compositions were supplied by the manufacturers according to their routine quality control data, as shown in Table 1. Quartz powder and quartz sand were supplied by Fengyang Chaoxin Building Materials Co., Ltd. (Anhui, China). Quartz powder featured a median particle size of about 3.7 μm, while quartz sand particles were approximately 30 μm, boasting a SiO_2_ content of 99.36%. A high-performance polycarboxylic acid-based powder served as the water-reducing agent, achieving a reduction rate of 30% or higher. The steel fibers used consist of copper-plated round and straight strands, measuring 13 mm in length and 0.2 mm in diameter, with an aspect ratio of 65. These fibers had a density of 7800 kg·m^−3^ and a tensile strength exceeding 2500 MPa. The superabsorbent polymer (SAP) was supplied by Yixing Kexin Chemical Co., Ltd. (Yixing, China) and appeared as white granules with a volume density ranging from 0.6 to 0.9 g·cm^−3^ and a water absorption capacity of 200 to 700 g·g^−1^, the particle size of the SAP ranged from 40 to 75 μm. Upon water absorption, SAP underwent a clear morphological transformation, as illustrated in Figure 1a,b. The absorbent aggregate ceramsite exhibited a porous internal structure, enabling significant water retention, The ceramsite had a bulk density of 832 kg·m^−3^, a water absorption rate of 12%, and a particle size ranging from 3 to 5 mm, and its macroscopic morphology is presented in Figure 1c. Laboratory tap water was used as the water source throughout the experiment.

### 2.2. Specimen Preparation

**Figure 2:**
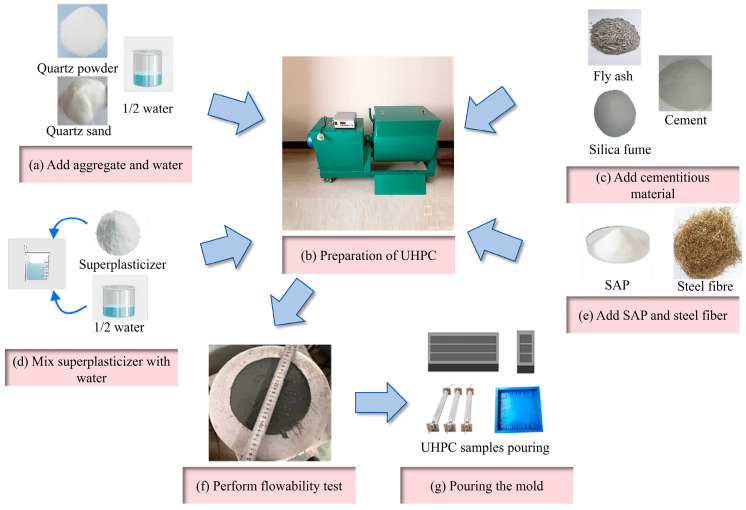
Specimen preparation process.

The preparation process of the UHPC specimens is outlined in Figure 2 and follows these steps:

(1) Begin by adding quartz sand and quartz powder to the mixer, along with half of the water. Stir for 2 min to ensure the quartz sand is evenly mixed with the water.
(2) Add all the gelling materials, including silica fume and fly ash, to the mixer and continue stirring at 60 rpm for 2 min to enhance dispersion.
(3) Dissolve the water-reducing agent in the remaining water, stir evenly, and gradually add this mixture into the mixer. Continue stirring for 4 min.
(4) Once the material transitions from powder to slurry, slowly add the prepared SAP, followed by sieving the steel fibers into the mixture. Stir for 2 min. Afterward, extract the UHPC slurry from the mixer, perform a fluidity test, and pour the mixture into the designated mold.
(5) For specimens intended for mechanical property testing, place them in a standard curing room (RH > 95%, 20 ± 2 °C) for 24 h after pouring. Demold the specimens and return them to the curing room until they reach the required specified age for testing.

It is important to note that specimens for drying shrinkage testing must be cured in the curing room for 2 days post-demolding before conducting the drying shrinkage test. For ceramsite specimens, add the ceramsite and quartz sand into the mixer at the start, maintaining the same process sequence thereafter. When preparing saturated SAP specimens, first extract the necessary additional water from the prepared water to saturate the SAP, and then add the saturated SAP to the mixer after the cementitious materials.

### 2.3. Mix Proportion of UHPC

Considering the w/b ratio in UHPC, as well as the dosage and addition method of SAP and ceramsite as the main variable design parameters, a total of 12 different groups of UHPC specimens were prepared; the mix ratios are shown in Table 2. The steel fiber content was fixed at 79 kg/m^3^, corresponding to a volume fraction of approximately 1.0%. The effective w/b ratio is defined as the ratio of the baseline mixing water to the mass of cementitious materials. The additional w/b ratio refers to the ratio of water introduced in addition to the baseline mixing water—including either externally added water or water supplied by internal curing materials—to the mass of cementitious materials. Accordingly, the effective w/b ratio is calculated as the total w/b ratio minus the additional w/b ratio. The total w/b ratio is defined as the ratio of the total water content of a given mixture to the mass of cementitious materials. All mixtures containing SAP or ceramsite were designed with a constant effective water-to-binder ratio of 0.18, which is identical to that of the reference mixture. The amount of additional water was carefully calculated to compensate for the water absorbed by the internal curing materials, thereby ensuring that the free water available for cement hydration remained consistent across all mixtures. Mixtures prepared with different water-to-binder ratios (0.18, 0.20, and 0.22) were used only as external reference groups.

### 2.4. Testing Methods

#### 2.4.1. Measurement of Water Absorption of SAP and Ceramsite

The water absorption capacity of internal curing materials was assessed using the tea bag method [29]. To simulate the chemical environment of the UHPC pore solution, tap water and three types of cementitious filtrates were prepared as testing media: cement (C), silica fume (SF), and fly ash (FA). These solutions were prepared with a water-to-binder (w/b) ratio of 5:1, and their specific proportions are detailed in Table 3. To prepare the filtrates, the water and cementitious materials were stirred for 5 min every hour over a 24 h period to ensure sufficient hydration while being covered to prevent carbonation. Subsequently, the mixtures were processed through a vacuum filtration system to obtain the clear filtrates. The rationale for selecting these multiple filtrates is that UHPC is a complex multi-component system; the high dosage of silica fume and fly ash significantly alters the ionic strength and concentration of multi-valent cations (e.g., Ca^2+^, Al^3+^) in the pore solution compared to pure cement paste. Since the swelling of SAP is highly sensitive to the osmotic pressure and ionic environment, testing in these specific media provides a more realistic estimation of the actual water absorption and release behavior within the UHPC matrix.

For the absorption test, a measured quantity of the dry material (*m*_1_) was placed in a pre-wetted tea bag (*m*_2_) and fully submerged in the prepared testing media. To minimize evaporation and carbonation, the beaker was quickly sealed with plastic wrap. At specified time intervals (1, 5, 10, 30, 60, 180, and 360 min), the tea bag was removed, and its surface was gently wiped with a dry cloth for approximately 30 s to eliminate excess liquid without compressing the sample. The mass of the tea bag with absorbed material (*m*_3_) was then weighed, and the sample was returned to the solution until the next measurement. Each measurement was repeated three times, and the average value was reported. The water absorption capacity (*A*) was calculated using Equation (1).

(1) $$A=\frac{m_{3}-m_{2}-m_{1}}{m_{1}}$$

In the formula, *m*_3_ represents the mass of the tea bag after the material has absorbed water, *m*_2_ is the mass of the pre-wetted tea bag, and *m*_1_ is the mass of the internal curing material before it was placed in the tea bag.

#### 2.4.2. Measurement of Flowability

The flowability of freshly mixed UHPC slurry was evaluated using the table-hopping method in accordance with the relevant specification [30]. Prior to testing, all tools in contact with the slurry were wiped clean. The slurry was then placed into the test mold in two layers, each layer being tamped to ensure uniform compaction. After leveling the excess slurry, the mold was lifted vertically, and the jumping table was operated for 25 drops. Finally, the average diameter of the spread slurry on the bottom surface was measured in two perpendicular directions to the nearest millimeter.

#### 2.4.3. Mechanical Property Testing

The mechanical performance of UHPC was evaluated in accordance with the relevant standard [31]. For each mix proportion, three prism specimens with dimensions of 40 mm × 40 mm × 160 mm were prepared. After casting, all specimens were cured under standard curing conditions (RH > 95%, 20 ± 2 °C) until the testing age of 28 days. After curing, flexural strength was first measured using a three-point bending test conducted on an MTS universal testing machine with a test span of 100 mm. The test was performed under displacement control at a loading rate of 1 mm/min. To ensure testing stability, a pre-load of 500 N was applied prior to loading. Three flexural strength tests were carried out for each mix proportion. Subsequently, compressive strength was determined using six specimens obtained from the fractured halves of the flexural test specimens, with a loading rate of 2.4 kN/s.

#### 2.4.4. Measurement of Autogenous Shrinkage and Internal Relative Humidity (IRH)

Early autogenous shrinkage was measured using an automatic shrinkage tester in accordance with ASTM C1698-2009 [32]. The testing system consisted of digital dial indicators, corrugated tubes, and circular steel pipes. The corrugated tubes were made of polyethylene, with a length of 420 ± 5 mm and inner and outer diameters of 25 mm and 30 mm, respectively. The wall thickness of the tubes was 0.5 ± 0.2 mm, and triangular corrugations were adopted to reduce lateral restraint effects. Considering the time required for specimen preparation and casting, the autogenous shrinkage test was initiated 1 h after casting. For each mixture, autogenous shrinkage measurements were conducted on three specimens, and the reported results represent the average values. The IRH of the specimens was monitored following the method described in the literature [28]. A DB485 temperature and humidity sensor, with a measurement range of 0–100% RH, was used for IRH monitoring. After pouring the UHPC slurry into the mold, a wooden stick was inserted to a depth of 50 mm to form a reserved hole. After setting, the stick was removed, and the sensor probe wrapped with gauze was inserted into the pre-formed hole. The specimen was then sealed with multiple layers of plastic wrap and aluminum foil to ensure airtight conditions. IRH data were recorded continuously using a computer, and the average IRH value of two specimens was reported.

#### 2.4.5. Measurement of Drying Shrinkage

The drying shrinkage test was conducted in accordance with Specification [33]. Specimens with dimensions of 25 mm × 25 mm × 280 mm were prepared, with three specimens for each mixture. Immediately after casting, the specimens were placed in a curing room maintained at a temperature of 20 ± 2 °C and a relative humidity exceeding 98%. The specimens were demolded after 1 day and then further cured for 2 days. The initial length was measured using a dial gauge and a length comparator. Subsequently, the specimens were transferred to a constant temperature and humidity chamber maintained at a temperature of 20 ± 2 °C and a relative humidity of 60 ± 5%. For each mixture, drying shrinkage measurements were conducted on three specimens, and the reported results represent the average values.

#### 2.4.6. Flat Plate Restraint Crack Resistance

**Figure 3:**
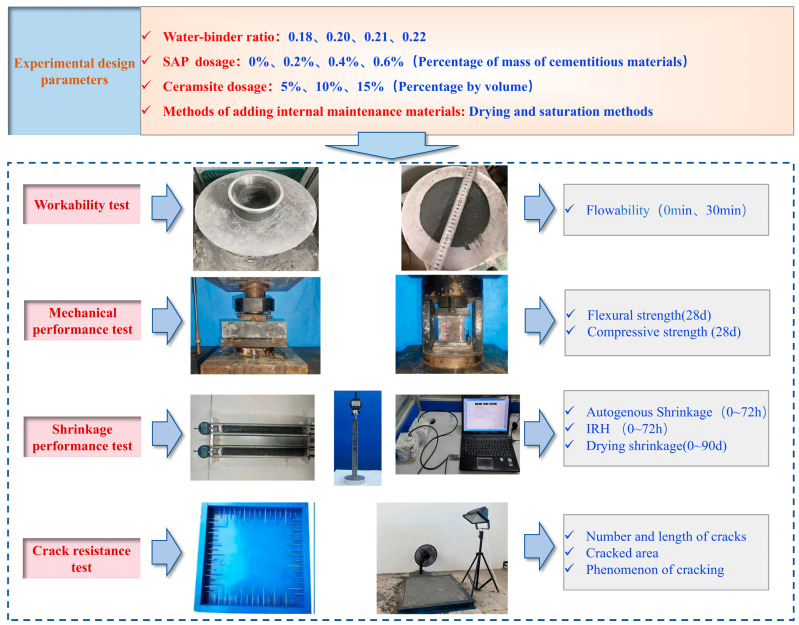
Specimen testing process.

The flat plate restrained cracking test was conducted using an anti-crack mold with dimensions of 600 mm × 600 mm × 63 mm, with reference to the test method specified in the relevant standard [34]. For each mixture, one representative flat plate specimen was prepared for the restrained cracking test, and the cracking tendency was evaluated comparatively under identical boundary conditions. After casting, a fan and a 1000 W iodine tungsten lamp were employed to simulate environmental conditions and accelerate the cracking process. The fan was positioned at an appropriate angle on the side of the mold to ensure a wind speed exceeding 5 m/s, directly acting on the surface of the UHPC specimen. Simultaneously, the iodine tungsten lamp supplied continuous and stable external heat radiation to the specimen surface to enhance evaporation and promote shrinkage cracking. Crack development was visually monitored at hourly intervals until the appearance of the first visible crack. After the first crack was observed, the specimen was continuously exposed to the restrained shrinkage conditions for a total duration of 72 h, after which the fan and lamp were turned off. At the end of the test, the total number of cracks on the UHPC specimen was recorded. The crack width was measured using a crack observer, and the crack length was determined using a ruler. The average crack area (a) was calculated according to Equation (2), the number of cracks per unit area (b) was calculated according to Equation (3), and the total crack area per unit area (C) was calculated according to Equation (4). The specific procedure for specimen testing is shown in Figure 3.

(2) $$a=\frac{1}{2N}\sum_{i}^{N}W_{i}L_{i}$$

(3) $$b=\frac{Ν}{A}$$

(4) C=a⋅b

where *Wi* is the maximum width of the *i*-th crack (mm), *Li* is the length of the *i*-th crack (mm), *N* is the total number of cracks, and *A* is the total area of the UHPC flat plate specimen (0.36 m^2^).

#### 2.4.7. SEM

After curing for 28 days, the UHPC specimens were fractured and immersed in anhydrous ethanol for 48 h to terminate the hydration process, followed by vacuum drying. The dried specimens were processed to a size of less than 10 mm. Microstructural observations were carried out using an IT-700HR scanning electron microscope at an accelerating voltage of 5 kV to examine the microscopic morphology and structural characteristics of the UHPC.

## 3. Results and Discussion

### 3.1. Water Absorption of SAP and Ceramsite

**Figure 4:**
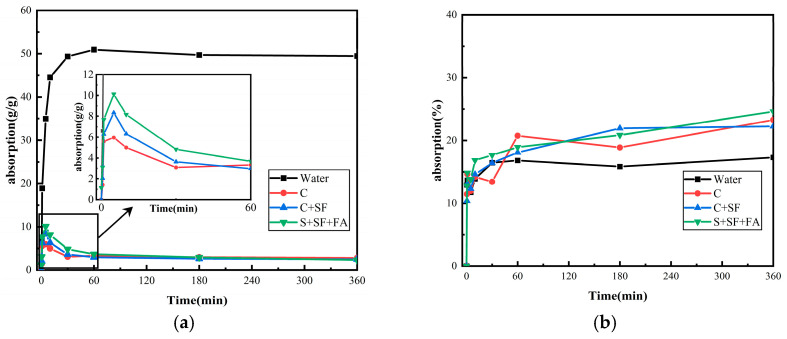
Changes in water absorption of SAP and ceramsite in tap water and filtrate: (**a**) SAP; (**b**) Ceramsite.

Figure 4 illustrates the water absorption and release behavior of SAP and ceramsite in four different solutions: tap water, cement filtrate, cement-silica fume filtrate, and cement-silica fume-fly ash filtrate. As shown in Figure 4a, SAP absorbs the most water in tap water, reaching 50 g/g after 60 min and remaining stable thereafter. In contrast, SAP shows significantly reduced absorption in cement-based filtrates, reaching saturation (2.4 g/g) within 10 min, followed by gradual water release. This behavior can be attributed to the ion content in the solutions. Previous studies indicate that the water absorption capacity of SAP is influenced by both ion concentration and total ion valence in the solution [35]. Higher ion concentrations reduce the osmotic pressure between the SAP and the surrounding solution, thereby decreasing the water absorption capacity of SAP. The high Ca^2+^ content in cementitious material filtrates causes SAP to achieve its maximum expansion rate within a short period, as it absorbs a substantial amount of Ca^2+^. This absorption increases the ion cross-linking density within the SAP, reducing its ability to bind water. Moreover, the water absorption rate of SAP in its absorbed state follows the order: C < C + SF < C + SF + FA. A slight increase in water absorption was observed with the inclusion of silica fume and fly ash. This is likely due to the supplementary cementitious materials lowering the Ca^2+^ concentration in the cementitious filtrate.

In contrast, ceramsite is a porous lightweight aggregate that absorbs water primarily through capillary action. As shown in Figure 4b, its absorption is relatively insensitive to ionic concentration, with minor differences observed between tap water and cementitious filtrates. Ceramsite attains an initial saturation state within 60 min, after which the absorption rate gradually decreases. The marginally higher uptake observed in cementitious solutions can be attributed to the increased solution density and ionic content. Overall, unlike SAP, the water absorption behavior of ceramsite is predominantly controlled by its physical characteristics—such as particle size, surface density, and pore structure—rather than by the chemical composition of the surrounding solution.

### 3.2. Flowability

**Figure 5:**
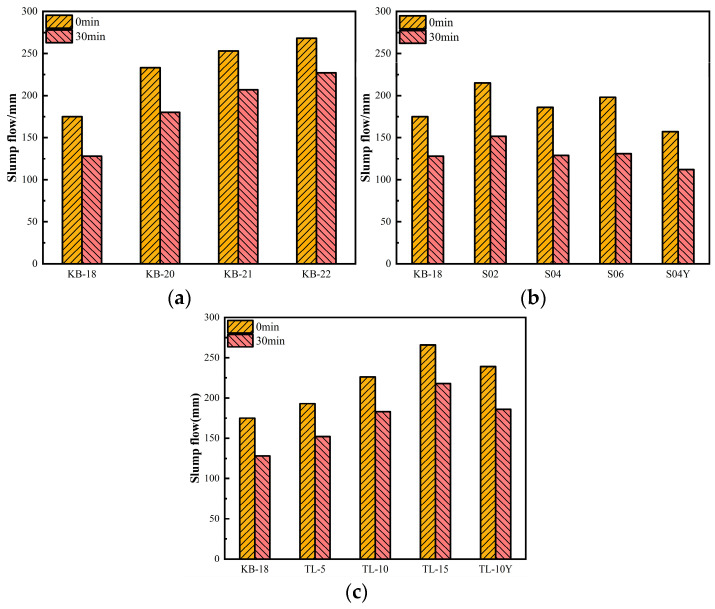
Changes in UHPC slump flow over time: (**a**) Water-binder ratio; (**b**) SAP; (**c**) Ceramsite.

Figure 5 shows the impacts of varying w/b ratio, dosage, and addition methods of SAP and ceramsite on the flowability of fresh UHPC. Figure 5a indicates that, when other parameters remain constant and the w/b ratio varies from 0.18 to 0.22, the slump flow of fresh UHPC progressively increases with the w/b ratio. After 30 min of standing, the slump flow decreased by approximately 50 mm on average, with the increase in w/b ratio having minimal impact on this phenomenon.

Figure 5b illustrates the nonlinear variation in UHPC flowability with increasing SAP dosage and corresponding increases in the w/b ratio. At SAP dosages of 0.2%, 0.4%, and 0.6%, with additional w/b ratios of 0.02, 0.03, and 0.04, respectively, the slump flow increased by 40 mm, 11 mm, and 23 mm relative to the control mixture. However, in comparison with Figure 5a, the incorporation of SAP generally resulted in a reduction in UHPC flowability, which is consistent with the findings reported by Liu et al. [36]. This behavior was attributed to the rapid water-absorption kinetics of SAP particles in the highly alkaline UHPC environment, which reduces the immediate availability of free water for particle lubrication. Furthermore, the pronounced decrease in slump flow observed in the pre-absorbed SAP groups, whereby fully swollen SAP particles increase the solid volume fraction and interparticle friction within the cementitious matrix. Figure 5c illustrates the effects of ceramsite dosage and addition method on the flowability of UHPC. At a constant cementitious material content and w/b ratio, the slump flow increases with increasing ceramsite content. The difference in slump flow between dry and pre-saturated ceramsite addition is relatively small, at approximately 13 mm. However, within 30 min after mixing, a slump flow reduction of 40–50 mm is observed, which is comparable to the flowability loss exhibited by UHPC mixtures containing SAP at varying w/b ratios. The porous structure of ceramsite results in a lower particle density, which may increase the risk of segregation under high-flowability conditions. To mitigate this effect, adjustments in admixture dosage—such as increasing the amount of water-reducing agent—may be required to ensure a uniform distribution of aggregates during mixing and casting.

### 3.3. Mechanical Performance

**Figure 6:**
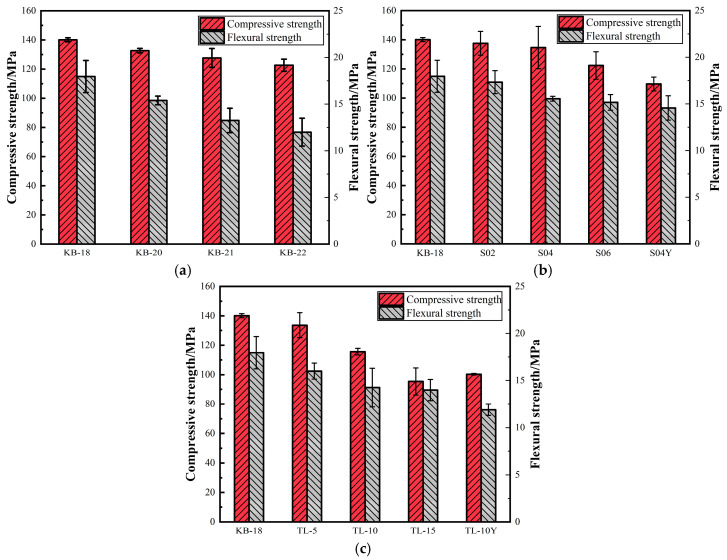
Changes in compressive strength and flexural strength of UHPC: (**a**) Water-binder ratio; (**b**) SAP; (**c**) Ceramsite.

The mechanical properties of the UHPC mixtures were evaluated, as shown in Figure 6. Figure 6a illustrates the effect of different w/b ratios on the mechanical properties of UHPC without internal curing materials. At a w/b ratio of 0.18, UHPC achieved a compressive strength of 140.1 MPa and a flexural strength of 17.96 MPa. An increase in the w/b ratio resulted in reductions in both compressive and flexural strengths. When the w/b ratio was increased by 0.04, yielding a total w/b ratio of 0.22, the compressive strength decreased by 12.4% to 122.67 MPa, while the flexural strength declined by 33% to 12 MPa. These results indicate that increasing the w/b ratio markedly deteriorates the mechanical performance of UHPC, with flexural strength being more sensitive than compressive strength. The superior strength of UHPC compared with ordinary concrete is primarily attributed to its dense microstructure and strong interfacial transition zone (ITZ) [37]. However, increasing the unit water content may weaken the ITZ, thereby reducing the mechanical properties of UHPC. Consistent with these findings, previous studies have demonstrated that the w/b ratio significantly affects cement hydration, porosity, and mechanical performance, with higher w/b ratios generally leading to lower strength [38].

The impact of SAP on the mechanical properties of UHPC is presented in Figure 6b. As the SAP dosage increases, a slight reduction in both compressive and flexural strengths is observed. Within the SAP dosage range of 0.2% to 0.6%, the compressive strength decreases from 137.57 MPa to 122.33 MPa. SAP dosages below 0.4% exhibit a negligible effect on strength, while a pronounced reduction in compressive strength occurs at a dosage of 0.6%. Although the incorporation of SAP slightly reduces the mechanical properties compared with the KB-18 group, the S02, S04, and S06 groups exhibit superior performance relative to the KB-20, KB-21, and KB-22 groups, at the same total w/b ratio. Specifically, the compressive strength increased by 3.7% at an SAP dosage of 0.2%, and the flexural strength increased by 12.6–26.4%, indicating that SAP can enhance the mechanical performance of UHPC under appropriate conditions. However, when the SAP dosage reaches 0.6%, the beneficial effect on compressive strength becomes negligible, which is attributable to the increased pore volume [39]. The increase in SAP dosage correlates with an improvement in flexural strength, likely due to enhanced bonding between the matrix and steel fibers. However, adding pre-absorbed SAP reduces the mechanical properties of UHPC. This reduction may be attributed to the larger particle size of pre-absorbed SAP, which leaves larger pores in UHPC after releasing water. Figure 6c illustrates the effect of ceramsite incorporation. At a ceramsite dosage of 5%, the compressive and flexural strengths reach 133.6 MPa and 16 MPa, respectively, representing an 11% reduction compared with the KB18 control. As the ceramsite dosage increases to 15%, both the compressive and flexural strengths decrease markedly to below 100 MPa and 13 MPa, respectively. This reduction can be attributed to microscopic defects induced by the relatively low strength of ceramsite compared with the UHPC matrix, as well as the possible uneven distribution of ceramsite particles. Overall, the addition of dry ceramsite leads to a reduction in both compressive and flexural strengths. Moreover, since ceramsite has a low density and tends to float, dry addition adversely affects its dispersion within the matrix. Therefore, it is recommended that ceramsite be pre-saturated with water prior to incorporation.

### 3.4. Autogenous Shrinkage and Internal Relative Humidity (IRH)

#### 3.4.1. Autogenous Shrinkage

**Figure 7:**
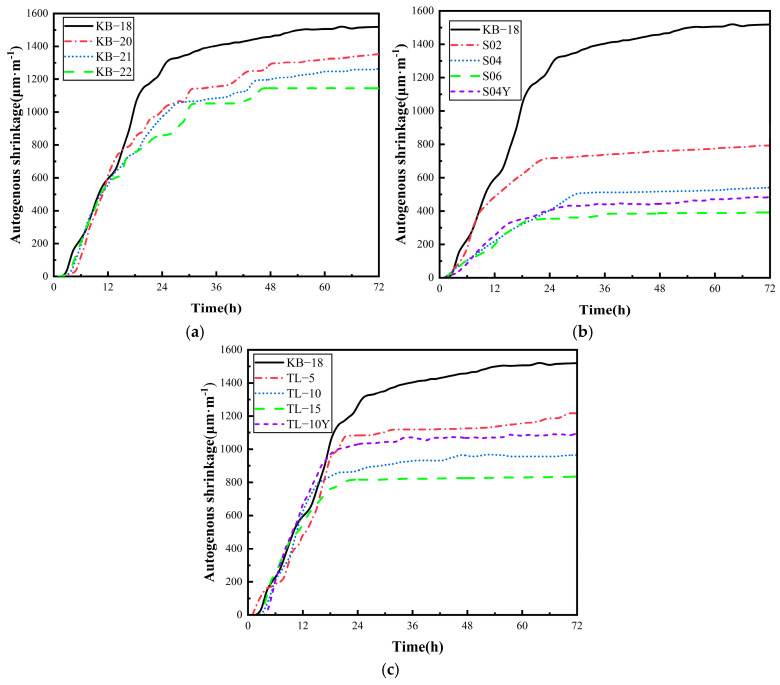
Changes in UHPC autogenous shrinkage over time: (**a**) Water-binder ratio; (**b**) SAP; (**c**) Ceramsite.

Figure 7 illustrates the early autogenous shrinkage behavior of UHPC for various mix ratios. As shown in Figure 7a, early autogenous shrinkage decreases as the w/b ratio increases. At a w/b ratio of 0.18, the autogenous shrinkage in 72 h is 1519 μm/m. However, in the KB-20, KB-21, and KB-22 groups, the 72 h autogenous shrinkage decreased by 10.9%, 16.8%, and 25%, respectively. In the initial stage of shrinkage, the water-cement ratio has little effect on the shrinkage rate.

Figure 7b illustrates that the addition of SAP significantly reduces the early autogenous shrinkage of UHPC. At SAP dosages ranging from 0.2% to 0.6%, the 72 h autogenous shrinkage values decrease by 48%, 64%, and 74%, respectively, compared with the KB-18 group. The effect of SAP on early autogenous shrinkage reduction exhibits a non-linear trend, with the shrinkage mitigation efficiency increasing as the SAP dosage increases. However, even at an SAP dosage of 0.6%, complete inhibition of early autogenous shrinkage remains difficult. In contrast, the pre-water-absorbed S04Y group exhibits only a 10% reduction in shrinkage. With an increase in ceramsite dosage from 0% to 5%, 10%, and 15%, the 72 h autogenous shrinkage values of UHPC decrease by 20%, 20%, and 13%, respectively. When the ceramsite dosage exceeds 10%, further increases diminish the effectiveness of autogenous shrinkage inhibition. At dosages below 10%, an approximately linear relationship is observed between ceramsite dosage and the reduction rate of autogenous shrinkage. The 72 h shrinkage value of the TL-10Y group is 1096 μm/m, corresponding to a 10% reduction compared with that of the TL-10 group, The observed enhancement in shrinkage mitigation for the TL-10 is supported by the findings of Tao Ji et al. [18]. Their study demonstrated that a higher prewetting degree of ceramsite ensures a more sustained internal water supply during the hardening phase, effectively compensating for the self-desiccation in the dense UHPC matrix.

#### 3.4.2. Internal Relative Humidity (IRH)

**Figure 8:**
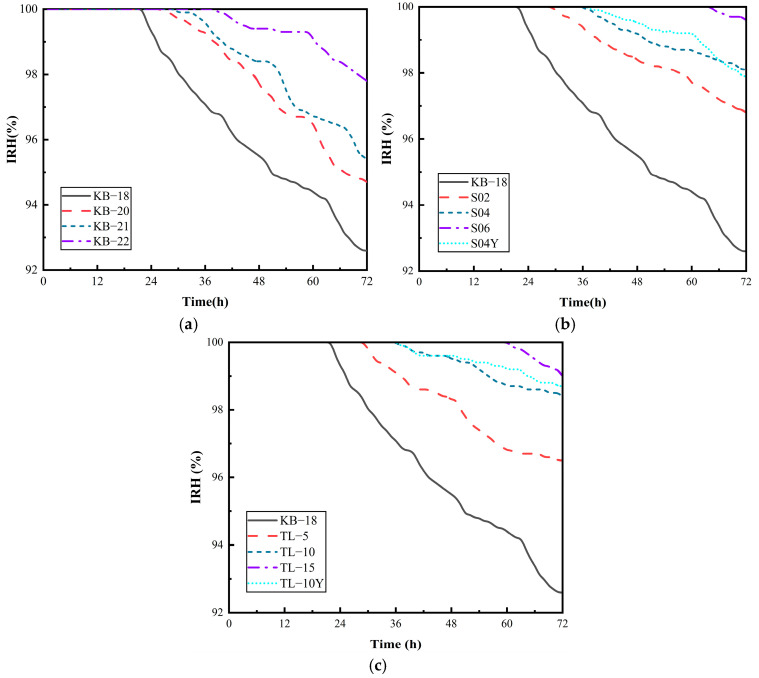
Changes in relative humidity inside UHPC over time: (**a**) Water–binder ratio; (**b**) SAP; (**c**) Ceramsite.

Autogenous shrinkage in UHPC primarily arises from the hydration of cementitious materials, inducing negative pressure within internal capillary pores, and is closely linked to the IRH of UHPC. Figure 8a shows the variations in IRH within the first 72 h after casting of UHPC across various w/b ratios. It can be observed that the IRH experienced a stable phase (approximately 100%) followed by a subsequent decline. The duration of stability extends from 22 h to 38 h with increasing w/b ratio, reaching a maximum IRH of 97.8%. Below a w/b ratio of 0.21, its effect on the IRH reduction rate is limited, and the rate of IRH decline starts to decelerate when the w/b ratio exceeds 0.22.

Figure 8b shows that increasing SAP dosage extends the IRH stable phase and reduces the rate of decline. At an SAP dosage of 0.6%, IRH remains at 100% for up to 65 h, though autogenous shrinkage still occurs due to early hydration heat. The SAP addition method has a minimal influence on early IRH behavior. SAP mitigates shrinkage by gradually releasing water, thereby maintaining a high IRH and reducing capillary tension, which becomes dominant once IRH drops below saturation. Figure 8c shows that higher ceramsite dosages also prolong IRH stability. Whether added in a saturated state or followed by external curing, ceramsite slows IRH loss and suppresses autogenous shrinkage due to its porous structure and internal curing capability.

### 3.5. Drying Shrinkage

**Figure 9:**
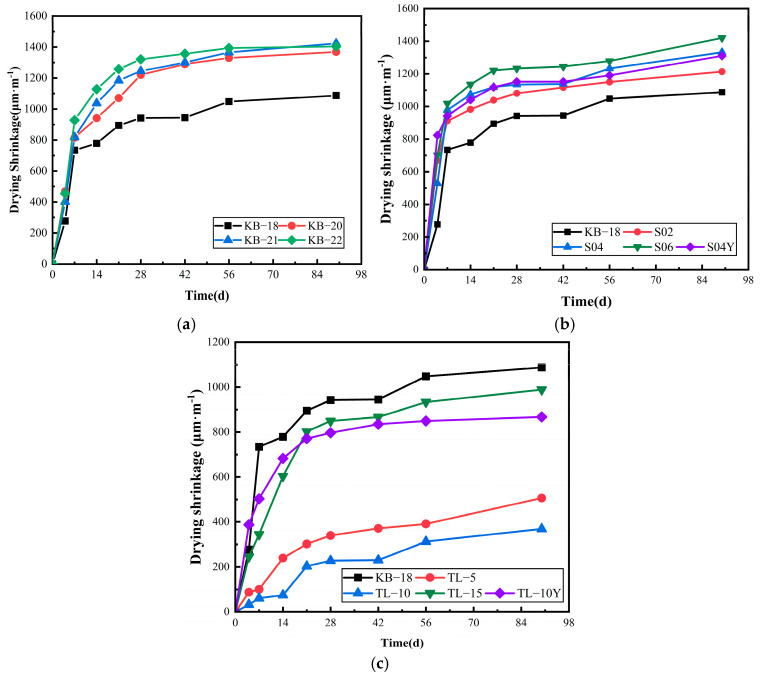
The change in UHPC drying shrinkage over time: (**a**) Water-binder ratio; (**b**) SAP; (**c**) Ceramsite.

Figure 9 presents the drying shrinkage behavior of UHPC at various mix ratios. As shown in Figure 9a, increasing the w/b ratio increases early-age shrinkage, particularly within the first 14 days, followed by steady progression. However, the final shrinkage of the KB-20, KB-21, and KB-22 groups at 90 days remains around 1400 μm/m, representing a 29% increase compared with the KB-18 group. This occurs because an increase in the w/b ratio leads to higher matrix porosity, facilitating water evaporation and loss, thereby increasing drying shrinkage.

Figure 9b shows that the addition of extra water via SAP further elevates shrinkage. At SAP dosages of 0.2%, 0.4%, and 0.6%, final shrinkage increases by 24%, 23%, and 31%, respectively, compared with KB-18. However, pre-absorbed SAP exhibits minimal impact, with both dry and pre-absorbed SAP showing similar shrinkage trends over time. Figure 9c shows that the incorporation of ceramsite reduces UHPC drying shrinkage. At 90 days, increasing the ceramsite volume from 5% to 15% results in shrinkage reductions of 53%, 66%, and 9%, respectively, compared to KB-18. This reduction is attributed to the internal curing effect of pre-saturated ceramsite, which helps maintain internal humidity. In contrast, unsaturated ceramsite yields only a 20% decrease, likely due to its low density and insufficient water absorption capacity, making it prone to floating and uneven distribution in the slurry, thereby reducing its effectiveness in inhibiting drying shrinkage. The drying shrinkage reduction of 66% achieved with 10% pre-saturated ceramsite at 90 days exceeds the reduction levels commonly reported for lightweight aggregates in conventional concrete, which are typically around 40% [26], highlighting the effectiveness of pre-saturated ceramsite as an internal curing agent in UHPC.

### 3.6. Shrinkage Crack Resistance Under Restraint

**Figure 10:**
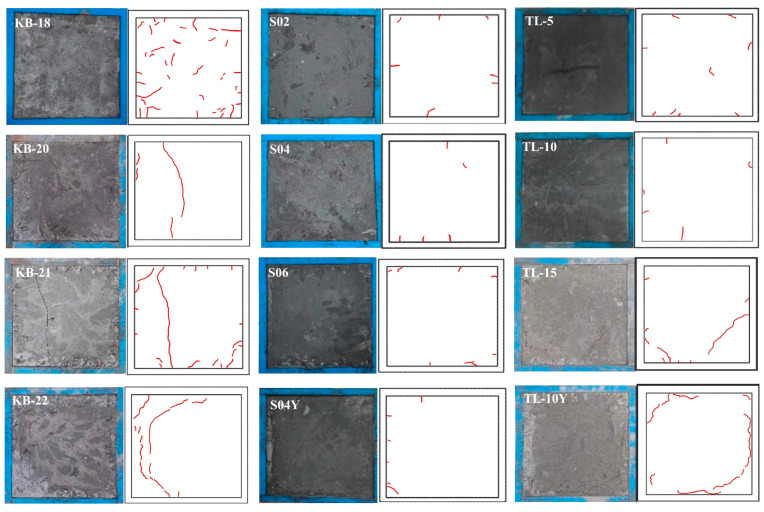
Crack situation of flat plate specimen.

**Figure 11:**
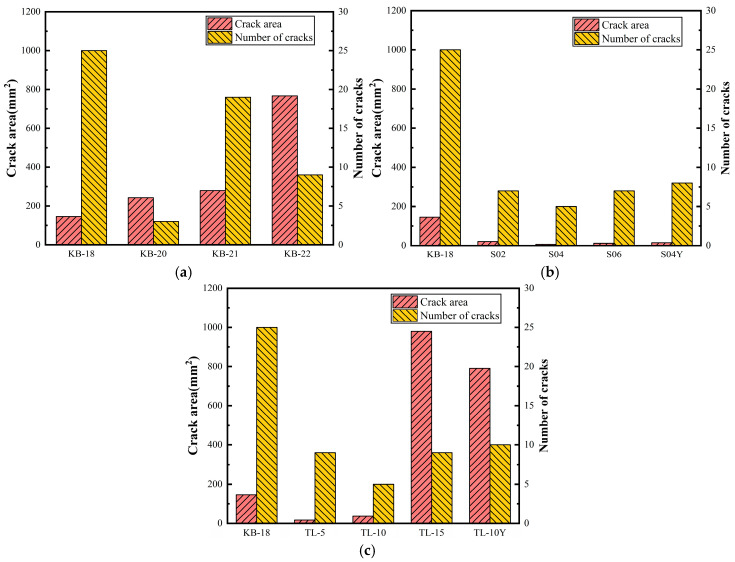
Crack area and number of cracks in UHPC flat specimens: (**a**) Water–binder ratio; (**b**) SAP; (**c**) Ceramsite.

Figure 10 and Figure 11 depict the cracking patterns, crack areas, and crack counts of UHPC specimens under flat plate restraint across varying w/b ratios. Cracks generally initiate from the angle steel at the panel edges and propagate toward the center. At a w/b ratio of 0.18, numerous fine cracks are concentrated along the plate edges. As the ratio increases to 0.20, the crack count decreases while crack size increases significantly. At 0.21, both the crack number and the dimensions of the primary cracks increase. At 0.22, the crack count further declines, but the maximum crack length and width increase, with the formation of continuous cracks observed. These results indicate that although higher w/b ratios reduce autogenous shrinkage, they do not improve cracking resistance. Instead, cracking becomes more severe, because an increase in the w/b ratio leads to a reduction in matrix strength and elastic modulus, which intensifies the settlement of steel fibers and alters the morphology of steel fibers at the crack interface [40], reduces strain capacity, and increases the tendency for larger deformations under identical restraint conditions.

At an SAP dosage of 0.2%, UHPC develops seven cracks within 72 h, each with a maximum width of 0.1 mm, a length of 6.2 cm, and a total area of 20.95 mm^2^. Increasing the dosage to 0.4% and 0.6% reduces crack counts to five and seven, with corresponding total crack areas of 7.2 mm^2^ and 12.35 mm^2^, respectively. Compared to KB-18, SAP significantly enhances crack resistance, maintaining crack numbers below 10 and the total crack area within 20% of the control across the SAP dosage range of 0.2–0.6%. The effect of pre-absorbed SAP on crack resistance is minimal. The improvement in crack resistance attributed to SAP is multifaceted, including supplying internal curing water to reduce shrinkage and pore pressure; enhancing cement hydration and matrix bonding, which increases internal restraint and tensile capacity; and the addition of SAP does not exacerbate UHPC expansion. Instead, it aids in achieving more uniform shrinkage reduction by incorporating additional water and redistributing steel fiber restraint stress.

When ceramsite is used as an internal curing agent, low dosages (<10%) are effective in reducing crack occurrence and crack length. At a ceramsite dosage of 5%, nine cracks are observed, with a maximum width of 0.1 mm and an average length of 5.2 cm. At 10% ceramsite, the crack count decreases to five, though the maximum width increases to 1 mm and the total area reaches 36.8 mm^2^. Both dosages exhibit good crack resistance. However, at a ceramsite dosage of 15%, cracking becomes severe, with a maximum crack length of 32 cm and a total area of 980 mm^2^, primarily concentrated at the specimen edges. Similar cracking behavior is observed with 10% dry ceramsite, though with greater severity. The excessive dosage likely increases UHPC fluidity and disrupts the distribution of steel fibers, thereby compromising crack resistance. To mitigate cracking, ceramsite should be pre-saturated with water, and its dosage should be limited to ≤10%.

### 3.7. SEM

**Figure 12:**
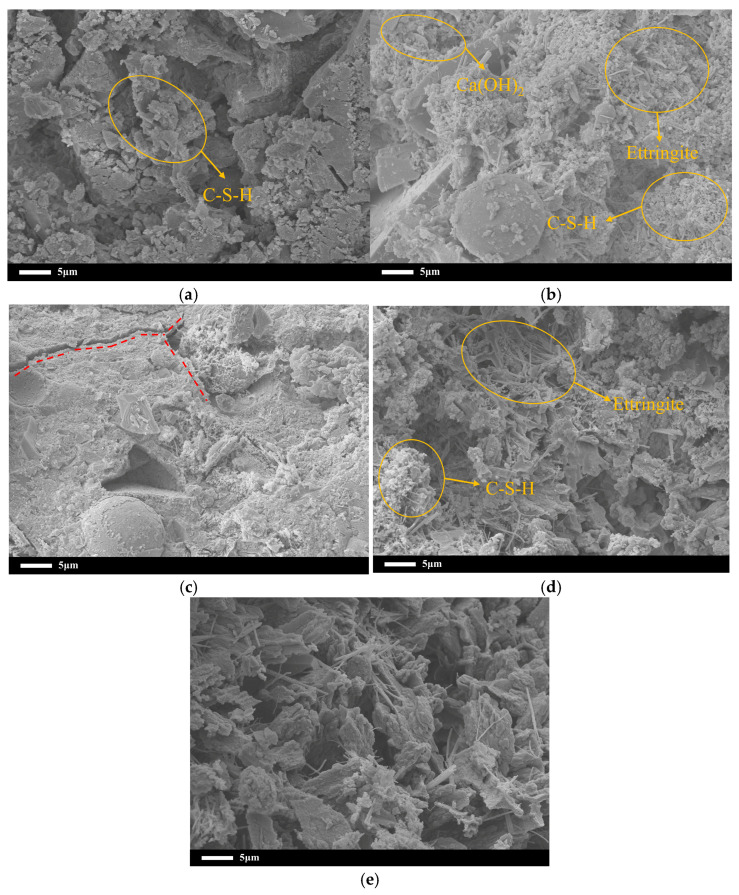
SEM microstructure of UHPC at 28d: (**a**) KB-18; (**b**) S04; (**c**) S04-Y; (**d**) TL-10; (**e**) TL-10Y.

Figure 12 shows the microscopic morphology of UHPC mixtures under different internal curing conditions at 28 days of age. Specimens exhibiting superior internal curing performance in the macroscopic tests were selected for microscopic analysis to elucidate the underlying mechanisms. As shown in Figure 12a, the KB-18 blank group sample shows a microstructure in which a large amount of flocculent calcium silicate hydrate (C-S-H) gel and a small amount of ettringite coexist. The calcium silicate hydrate gel is relatively loosely distributed, the mortar structure is loose, the connection between the hydration products is not tight enough, and there are many pores.

Compared with the blank group, the microstructure of the S04 and S04Y samples has changed significantly. Not only were numerous needle-shaped ettringite crystals observed, but irregular flaky particles morphologically resembling calcium hydroxide (Ca(OH)_2_) were also observed. This phenomenon indicates that the additional moisture introduced by the internal curing material effectively promotes the hydration process of the cementitious material and generates more abundant C–S–H gel. These gels are intertwined, which significantly improves structural density, making the mortar structure more compact and reducing porosity. Further observation of the S04Y sample revealed the presence of a certain number of microscopic cracks within the matrix, as indicated by the red dotted lines in Figure 12c. The formation of these cracks can be attributed to the volume shrinkage of SAP during water release, which leads to the formation of pores. If the microstructural damage caused by SAP is not filled or repaired by subsequent hydration products in the plastic stage, the internal shrinkage stress within the slurry will readily induce crack propagation [41]. Therefore, during the SAP internal curing process, attention should be paid to the potential adverse effects on the microstructure. In contrast, a certain amount of hydration products and C–S–H gel were also observed in the TL-10 and TL-10Y samples. The density of their internal structure was significantly lower than that of the specimens containing SAP, and pores could still be clearly identified within the specimens. The inherently porous structure of ceramsite may have affected its internal curing efficiency to a certain extent, and its water absorption capacity is relatively limited. However, compared with the blank group, the internal pore distribution of UHPC is still optimized to some extent.

Although a direct quantitative pore structure analysis was not conducted in this study, the observed mitigation of shrinkage and enhancement of crack resistance can be qualitatively attributed to pore structure modification induced by internal curing [42]. The incorporation of SAP and pre-saturated ceramsite promotes continuous hydration, which potentially reduces capillary pore connectivity and refines the pore structure. This interpretation is supported by the SEM observations, which reveal denser hydration products and fewer microstructural defects in mixtures with effective internal curing. Such pore structure refinement is known to alleviate capillary tension and internal moisture gradients, thereby contributing to reduced autogenous shrinkage and restrained cracking.

## 4. Conclusions

The effects of water-to-binder ratio, internal curing material type (SAP and ceramsite), dosage, and addition method on the fresh and hardened properties of UHPC were systematically investigated. The water absorption behavior of internal curing materials, as well as the flowability, mechanical performance, shrinkage behavior, and restrained cracking resistance of UHPC, were experimentally evaluated. Based on the obtained results, the following conclusions can be drawn:

(1) Increasing the w/b ratio results in varying degrees of deterioration in the mechanical properties of UHPC. Under an identical total w/b ratio, the incorporation of SAP as an internal curing material can slightly enhance mechanical performance, whereas a ceramsite content exceeding 10% leads to a pronounced reduction in both compressive and flexural strengths.
(2) At a constant effective w/b ratio, both SAP and ceramsite effectively mitigate autogenous and drying shrinkage of UHPC. Their influences on autogenous shrinkage are comparable; however, owing to its fine aggregate characteristics and internal water storage capacity, ceramsite exhibits a more pronounced inhibitory effect on drying shrinkage.
(3) SAP and pre-saturated ceramsite with a volume content of less than 10% can significantly reduce autogenous shrinkage while enhancing the restrained cracking resistance of UHPC. In contrast, excessive ceramsite dosage (>10%) or dry addition methods, although capable of reducing shrinkage, adversely affect crack resistance due to phenomena such as aggregate floating and fiber redistribution, leading to fewer but wider cracks.
(4) The addition of internal curing materials promotes cement hydration, increases the formation of AFt and C-S-H gel, refines the pore structure, and enhances matrix compactness, thereby improving the shrinkage resistance and cracking performance of UHPC.
(5) Considering the combined effects on mechanical properties, shrinkage mitigation, and crack resistance, it is recommended that SAP be incorporated in a dry state with an optimal dosage of 0.4% by mass of cementitious materials. For ceramsite, pre-saturation prior to mixing is essential, and the recommended dosage is approximately 5% by volume.

Despite the insights provided by this study, several limitations should be acknowledged. The investigated internal curing materials were limited to specific SAP and ceramsite types, and the influences of different polymer chemistries, particle sizes, and lightweight aggregate properties were not considered. In addition, the shrinkage and restrained cracking behavior was mainly evaluated under laboratory-controlled conditions and at early to medium ages, which may not fully represent the complex environmental and restraint conditions encountered in practical engineering applications. Moreover, the microstructural analysis was primarily qualitative in nature. Future work should focus on exploring a wider range of internal curing materials and parameters, evaluating long-term shrinkage and durability performance, and validating the proposed internal curing strategies under more realistic restraint conditions and at larger structural scales.

## Figures and Tables

**Table 1:** Chemical composition of cement and additives.

Materials	SiO_2_ (%)	Al_2_O_3_ (%)	Fe_2_O_3_ (%)	CaO (%)	MgO (%)	SO_3_ (%)	Loss of Ignition (%)
Cement	25.56	6.38	4.04	63.62	1.25	2.01	3.02
Silica fume	96.15	1.06	1.44	0.46	0.82	-	3.92
Fly ash	45.1	24.2	5.54	5.60	2.63	2.10	3.19

**Table 2:** Composition of UHPC mixtures (kg/m^3^).

	Cement and Additives			Quart Powder	Quart Sand	Steel Fiber	Superplasticizer	Ceramsite	SAP	Total w/b	Effective w/b	Additional w/b
Labels	Cement	Silica Fume	Fly Ash									
KB-18	624	156	62.4	156	926	79	16.85	0	0	0.18	0.18	0
KB-20	624	156	62.4	156	926	79	16.85	0	0	0.20	0.18	0.02
KB-21	624	156	62.4	156	926	79	16.85	0	0	0.21	0.18	0.03
KB-22	624	156	62.4	156	926	79	16.85	0	0	022	0.18	0.04
S02	624	156	62.4	156	926	79	16.85	0	1.69	0.20	0.18	0.02
S04	624	156	62.4	156	926	79	16.85	0	3.37	0.21	0.18	0.03
S06	624	156	62.4	156	926	79	16.85	0	5.07	0.22	0.18	0.04
S04Y	624	156	62.4	156	926	79	16.85	0	3.37	0.21	0.18	0.03
TL-5	624	156	62.4	156	926	79	16.85	74	0	0.186	0.18	0.006
TL-10	624	156	62.4	156	926	79	16.85	148	0	0.193	0.18	0.013
TL-15	624	156	62.4	156	926	79	16.85	222	0	0.20	0.18	0.02
TL-10Y	624	156	62.4	156	926	79	16.85	148	0	0.193	0.18	0.013

Note: KB represents specimens without internal curing materials, S represents specimens with SAP added as internal curing materials, TL represents specimens with ceramsite added as internal curing materials. S04Y indicates the SAP is saturated with water in advance, and TL-10Y indicates the pre-dried treatment of lightweight aggregate.

**Table 3:** Mixed solution proportions.

Labels	Cement (%)	Silica Fume (%)	Fly Ash (%)	Water (%)
Water	0	0	0	100
C	16.7	0	0	83.3
C + SF	13.3	3.4	0	83.3
C + SF + FA	12.4	3.1	1.2	83.3

## Data Availability

The original contributions presented in this study are included in the article. Further inquiries can be directed to the corresponding author.
